# Enhanced recovery care versus traditional non-ERAS care following osteotomies in developmental dysplasia of the hip in children: a retrospective case-cohort study

**DOI:** 10.1186/s12891-020-03243-z

**Published:** 2020-04-13

**Authors:** Jin Li, Saroj Rai, Renhao Ze, Xin Tang, Ruikang Liu, Pan Hong

**Affiliations:** 1 Department of Orthopaedic Surgery, Union Hospital, Tongji Medical College, Huazhong University of Science and Technology, Wuhan, 430022 China; 2 Department of Orthopaedics and Trauma Surgery, National Trauma Center, National Academy of Medical Sciences, Mahankal, Kathmandu, Nepal; 3 First School of Clinical Medicine, Tongji Medical College, Huazhong University of Science and Technology, Wuhan, China

**Keywords:** Enhanced recovery after surgery, Developmental dysplasia of the hip, Complications, Hospitalization, Osteotomy

## Abstract

**Background:**

Enhanced recovery after surgery (ERAS) has been shown to shorten the length of hospital stay and reduce the incidence of perioperative complications in many surgical fields. However, there has been a paucity of research examining the application of ERAS in major pediatric orthopaedic surgeries. This study aims to compare the perioperative complications and length of hospital stay after osteotomies in children with developmental dysplasia of the hip (DDH) between ERAS and traditional non-ERAS group.

**Methods:**

The ERAS group consisted of 86 patients included in the ERAS program from January 2016 to December 2017. The Control group consisted of 82 DDH patients who received osteotomies from January 2014 to December 2015. Length of hospital stay, physiological function, postoperative visual analogue scale (VAS) score, and postoperative complications were compared between the two groups.

**Results:**

The mean duration of hospital stay was significantly reduced from 10.0 ± 3.1 in the traditional care group to 6.0 ± 0.8 days in the ERAS(*P* < 0.001). The average VAS score in the first 3 days was significantly lower in the ERAS group (2.9 ± 0.8) than the traditional non-ERAS group (4.0 ± 0.8) (*P* < 0.001). However, there was no significant difference in the frequency of break-out pain (VAS > 4) between two groups (29.5 ± 6.3 times vs.30.6 ± 6.5 times, *P* = 0.276). The frequency of postoperative fever was lower in the ERAS group. The frequency of urinary tract infection in both groups were not noticeable because the catheter was removed promptly after the surgery.

**Conclusion:**

The ERAS protocol is both safe and feasible for pediatric DDH patients undergoing osteotomies, and it can shorten the length of hospital stay without increasing the risk of perioperative complications.

## Background

Minimal invasive surgery (MIS), damage control theory (DCT), and enhanced recovery after surgery (ERAS) are three breakthroughs in the twenty-first century [1]. ERAS was proposed by Henrik Kehlet in the 1990s [2], and it has been widely applied in many surgical specialties for more than 20 years. The ERAS protocol is an evidence-based multidisciplinary perioperative approach, aiming to reduce the hospital stay, decrease the incidence of surgery-related complications, and promote early rehabilitation [3]. In orthopaedic surgery, ERAS was adopted in high-volume standard procedures, such as primary hip and knee replacements. There are reports that the ERAS protocol is both safe and effective in orthopaedic surgeries [4–6]. A meta-analysis showed that ERAS could facilitate the rapid recovery of physiological function, reduce the incidence of perioperative complications, shorten the duration of hospital stay [7].

However, there has been a paucity of literature examining the application of ERAS in major orthopaedic surgeries in children.

Developmental Dysplasia of the Hip (DDH) is a common bone and joint deformity of lower limbs in the pediatric population [8]. The incidence of DDH is 1.5 to 2.5 cases per 1000 live births [9, 10]. The DDH treatment aims to achieve concentric hip reduction and painless hip joint in the adolescence and adulthood. Pavlik harness is an effective option for children younger than 6 months with DDH [9, 11, 12]. However, if diagnosed at walking age, the treatment strategy might be complex and controversial [12, 13].

Pelvic osteotomy (PO) with or without proximal femoral osteotomy (PFO) is the mainstream surgical treatment for DDH [14]. The choice of osteotomy is mainly based on the age at the time of surgery, pathological and anatomical characteristics, severity of the dislocation, and clinical experience and preference of the surgeon. Osteotomy is the only effective method of treatment of DDH in patients older than 18 months [15]. However, it is not without complications. The most common perioperative complications include infection, hematoma, wound dehiscence and pain, leading to an extended hospital stay. Thus, establishing an ERAS protocol is urgent for major orthopaedic surgeries in children. Since DDH is the most commonly performed elective surgery in our hospital, we firstly established and implemented an ERAS protocol for DDH in 2016 [16]. Before the establishment of the ERAS protocol, the traditional care (non-ERAS) plan was implemented for DDH patients. The specific requirement for each step was not included in the traditional non-ERAS care plan.

This retrospective study aims to compare the incidences of complications and length of hospitalization after osteotomies in children with DDH between the ERAS group and the traditional non-ERAS group. To our knowledge, this is the first study to evaluate the effectiveness of an ERAS protocol for DDH osteotomies in children.

## Methods

### ERAS program

Based on the ERAS protocol adopted in the pediatric surgery and orthopaedics department, an ERAS protocol for pediatric orthopaedic surgery was developed following a series of discussions with experts from pediatric orthopaedic surgery, anesthesiology, pediatrics, and nurses from both the ward and operating room (OR). The protocol was then implemented for DDH patients undergoing surgical treatment from January 1, 2016.

The ERAS protocol for DDH osteotomies surgery included the following elements (Tables 1 and 2): preoperative education and counselling, antimicrobial prophylaxis, multimodal analgesia, postoperative nausea and vomiting (PONV) prophylaxis, early oral food intake, less daily intravenous infusion volume, early removal of wound drainage and urinary catheters, early mobilization, and discharge criteria. Preoperative education and counselling included the aim and procedures of the ERAS protocol, pain coping strategies, the parental expectation of this surgery, discharge criteria, and a follow-up plan. The fasting guideline requires cessation of clear fluid for at least 2 h as well as solid foods for 6 h before anaesthesia [17]. The temperature of the operative room was controlled at 24 degrees, and warm fluids were used. Patients were administered with multimodal analgesia postoperatively including local infiltration of ropivacaine (2.0 mg/ml) around the incision, analgesia infusion pump based on the parental requirements, intravenous nonsteroidal anti-inflammatory (flurbiprofen, 2 mg/Kg) for 3 days, and then oral dexibuprofen oral suspension if necessary. The regular diet was advised after the patient fully recovered from the anaesthesia. A lower intravenous infusion volume was defined as less than 300 ml/d after surgery. The Foleys catheter was removed within 24 h postoperatively. The drainage tube was recommended to be removed within 48 h postoperatively. Early mobilization, including the on-bed movement of the healthy leg and upper extremities on postoperative day (POD) 1. The antithrombotic prophylaxis was not necessary since children were at low risk of deep venous thrombosis (DVT). Discharge criteria were as follows: a visual analogue scale (VAS) score of < 3 points with or without the use of oral analgesia; normal diet, no need for intravenous fluid; normal body temperature, no evidence of wound infection with normal complete blood count and C-reactive protein level; and no serious complications. We encouraged patient discharge once the discharge criteria were met.

**Table 1:** The ERAS protocol versus traditional care

	Non-ERAS Care protocol	ERAS protocol
Psychological counseling	Informed consent	Education and illustration
Nutrition and Anemia Evaluation	No requirement	Proper assessment
Preoperative fasting	Fasting 12 h, water 4 h	Fasting 6 h, water 2 h
PONV prophylaxis	No requirement	5-HT receptor antagonist
Postoperative diet	6 h	Regular diet after anesthesia awareness
Perioperataive Analgesic management	NSAIDs	Comprehensive analgesic regimen
Removal of urinary catheter	No requirement	POD1
Removal of wound drainage	POD 2–3	POD 2

**Table 2:** Components of ERAS pathway for DDH

Preoperative	Intraoperative	Postoperative
Parental education	Local anesthesia of incision	Analgesia infusion pump
Evaluation of nutrition	Maintenance of body temperature	Early diet after anesthesia awareness
No bowel preparation	Maintenance of blood volume	PONV prophylaxis
No prolonged fasting		Early removal of catheters
Preventive antibiotics		Compliance and follow-up
Carbohydrate loading		

### Population

The hospital medical records of DDH patients receiving unilateral osteotomies in our institution were retrospectively reviewed. We included 82 patients in the traditional non-ERAS group or control group who underwent DDH surgery just prior to the implementation of the ERAS protocol (from January 2014 to December 2015). The ERAS group consisted of 86 patients who underwent DDH surgery after the implementation of the ERAS protocol (from January 2016 to December 2017).

The inclusion criteria were: 1) Patients aged between 1.5 years and 6 years received DDH surgery (PO + PFO) after failed nonoperative management, and 2) Unilateral single-stage surgery for bilateral DDH. The exclusion criteria were: 1) Patients with prior neuromuscular disease, cerebral palsy, scoliosis and hyperlaxity, and 2) Patients over the age of 6 years.

This study is approved by the Ethics Committee of Tongji Medical College, Huazhong University of Science and Technology.

### Surgical techniques

**Fig. 1:**
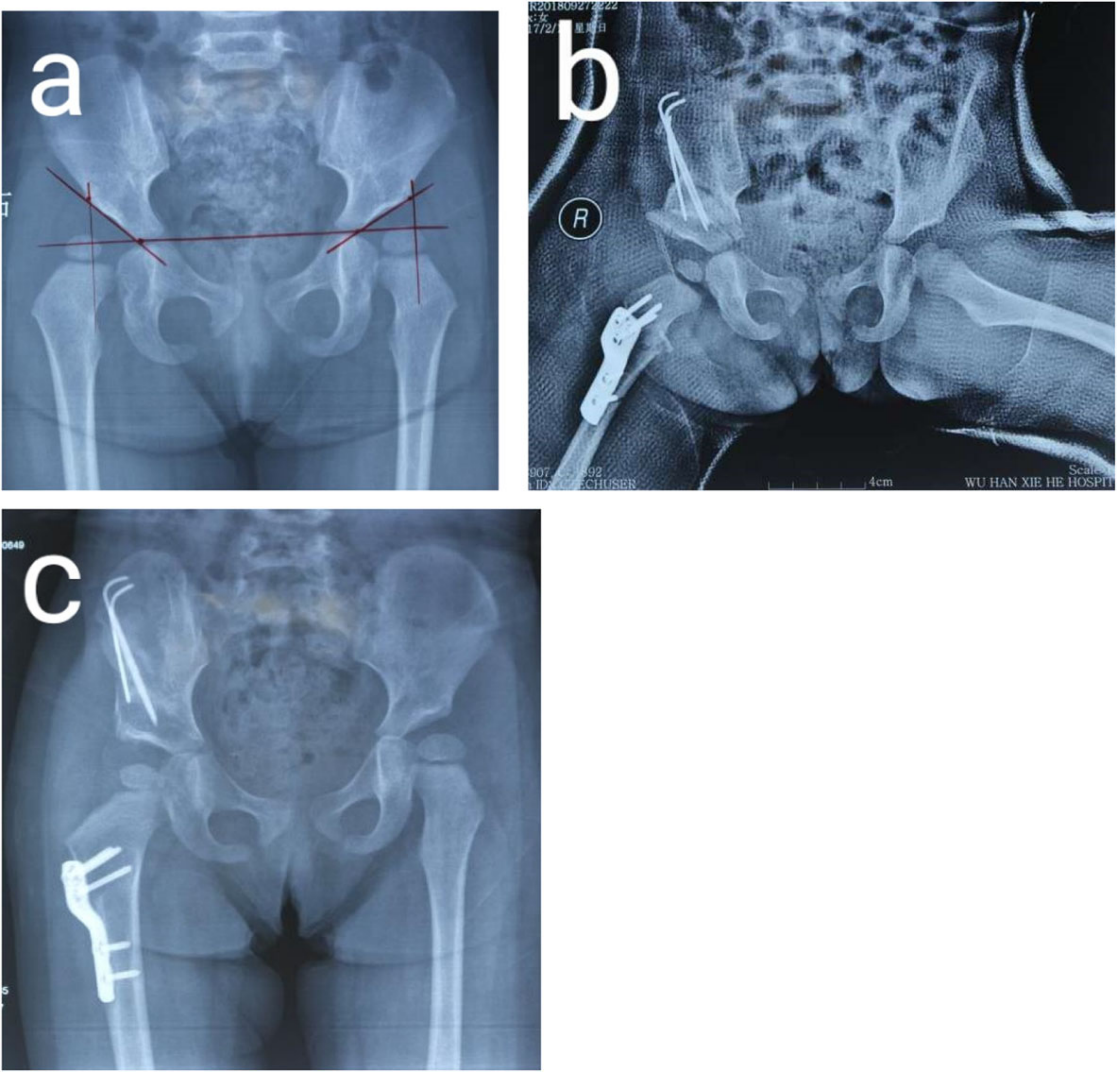
a 2 and half year-old girl received PO + PFO. **a** AP view of the pelvis before the surgery. **b** AP view of the pelvis after the surgery. **c** AP view of the pelvis at 3 months follow-up visit

All the patients underwent PO + PFO (See Fig. 1). Pelvic osteotomy (PO) is meant for increasing the acetabular coverage on the femoral head. Acetabuloplasty offers a higher rate of correction of acetabular dysplasia in comparison with reorientation osteotomies [18].

Pemberton osteotomy has been a preferred method of pelvic osteotomy in our institution. Proximal femoral osteotomies (PFO) are designed to reorient the femoral head by derotation and increasing the varus in order to stabilize and stimulate acetabular development [19, 20]. Pediatric hip plate (PHP) system is used to fixate the osteotomy at the femoral side.

After the surgery, all patients were immobilized in Spica cast for 4–6 weeks until the first out-patient visit.

### Clinical evaluation variables

Physiological function outcome indicators, such as the first time that the patient was able to eat, defecate, as well as the removal time of the Foleys catheter and drainage tubes, and duration of hospital stay were recorded.

The postoperative pain response after surgery was recorded, including the average VAS score, the highest VAS score, and the frequency of break-out pain (VAS ≥ 4). The incidence of all postoperative complications, including nausea, vomiting and fever, were recorded.

### Statistical analysis

All descriptive data were presented as the mean ± SD. Statistical analysis was performed using SPSS (SPSS Inc., Chicago, IL). A *p*-value of < 0.05 is regarded as statistical significance.

## Results

### Demographic data

Demographic data are shown in Table 3. There were no significant differences in age, sex, surgical duration, intraoperative blood loss, and other comorbidities between the ERAS group and traditional non-ERAS group.

**Table 3:** Demographic characteristics

Category	Non-ERAS Care (*n* = 82)	ERAS (*n* = 86)	*P* Value
Average age	4.2 ± 1.3	4.0 ± 1.3	0.515
Gender			
Male	13	11	
Female	69	75	0.358
BMI (mean)	23.28 ± 1.45	23.62 ± 1.45	0.138
Operative time (min)	106.4 ± 8.7	104.2 ± 8.9	0.148
Intraoperative bleeding (ml)	144.1 ± 28.1	146.9 ± 28.6	0.538

*BMI* body mass index

### Clinical outcomes

The removal of Foleys and drainage tubes were significantly earlier in the ERAS group than that in the traditional non-ERAS group (Table 4). The mean duration of hospital stay was significantly reduced from 10.0 ± 3.1 in the traditional non-ERAS group to 6.0 ± 0.8 days in the ERAS(*P* < 0.001).

**Table 4:** Postoperative physiological function outcomes

	Non-ERAS Care	ERAS	*p* value
First eating time, h	12.2 ± 1.5	5.2 ± 0.8	< 0.001
Urinary catheter removal, h	54.2 ± 3.8	10.0 ± 1.5	< 0.001
Wound drainage removal, h	41.9 ± 3.7	29.3 ± 3.6	< 0.001
First defecation,h	4.4 ± 1.1	4.4 ± 1.1	0.869
Length of hospital stay, d	10.0 ± 3.1	6.0 ± 0.8	< 0.001

The average VAS score in the first 3 days was better in the ERAS group than the traditional non-ERAS group (2.9 ± 0.8 vs. 4.0 ± 0.8, P < 0.001), and maximum VAS score (4.1 ± 0.8 vs. 4.9 ± 0.8, P < 0.001) (Table 5). However, there was no significant difference in the frequency of break-out pain (VAS > 4) between two groups (29.5 ± 6.3 vs.30.6 ± 6.5, *P* = 0.276).

**Table 5:** VAS pain score of average first 3d after surgery

	Non-ERAS Care	ERAS	*p* value
Mean VAS score	4.0 ± 0.8	2.9 ± 0.8	< 0.001
Maximum VAS score	4.9 ± 0.8	4.1 ± 0.8	< 0.001
Break-out pain (VAS ≥ 4)	30.6 ± 6.5	29.5 ± 6.3	0.276

Table 6 shows the surgery-related complications in both groups. There were no serious complications observed in this study. PONV was more common in the ERAS group as we used opiates as analgesics in the infusion pump. However, fever was less in the ERAS group, and no patients had postoperative urinary tract infection.

**Table 6:** Postoperative complications and adverse reactions

	Non-ERAS Care (*n* = 82)	ERAS (*n* = 86)	*p* value
Nausea and vomiting	38 (46.3%)	63 (73.3%)	< 0.001
Fever	1.5 ± 1.1	1.0 ± 0.9	0.002
Urinary infection	0	0	0.663

### Compliance with ERAS core components

Compliance with the core components of the ERAS protocol (Table 2) is shown in Table 7. During the perioperative period, the fasting time before surgery was shortened in the ERAS group. The application of local analgesia, oral analgesia, PONV prophylaxis was well executed in the ERAS group. Because of the possible adverse reactions of opiates (Sufentanil or Oxycodone), the application of analgesia infusion pump was not well executed. In all patients, oral Dexibuprofen was given whenever necessary. Compliance of early removal of the Foleys catheter and drainage tubes in the ERAS group was 100 and 95.3% respectively. The most common cause of delayed removal of drainage catheters was high drainage volume on POD2, which may increase the risk of postoperative hematoma and local infection. Compliance with early regular diet was 95.3%.

**Table 7:** Compliance with ERAS core components in ERAS group

	No. following protocol	N%
Preoperative education and counseling	86	100%
Preoperative fasting 6 h, clear water 2 h	86	100%
Multimodal analgesia		
Local anesthesia of incision	86	100%
Analgesia infusion pump	65	75.6%
Scheduled intravenous analgesia	86	100%
Oral analgesia	86	100%
PONV prophylaxis	86	100%
Early diet	82	95.3%
Removal of Urinary catheter on POD 1	86	100%
Removal of wound drainage on POD2	82	95.3%

## Discussion

The most important finding of the study was that the application of ERAS protocol in children undergoing osteotomies for DDH resulted in a reduced hospital stay and better pain tolerance without a significant increase in perioperative complications.

ERAS protocol has been widely applied in many surgical fields, especially in abdominal surgery. In the past few years, there were reports on its application in orthopaedic surgery such as joint replacement, trauma and spine surgery [21–23]. However, the literature on pediatric orthopaedic surgery was quite rare. A pediatric population is a group that requires special attention and discretion.

Preoperative evaluation of malnutrition and anaemia are critical as they are associated with a higher rate of postoperative complications such as delayed wound healing, infection, and prolonged duration of hospital stay [24, 25]. A recent history of common cold, cough, diarrhoea should be taken carefully before admission; if one had such symptoms, surgery should be postponed. A preoperative haemoglobin level of less than 12 g/dL should be investigated to rule out other pathological causes. If the bodyweight is 2 standard deviations lighter than the standard weight in peers, more attention should be paid to the patients.

Preoperative counselling and education are crucial components of the ERAS protocol. Patients and patient’s legal guardians should be counselled and educated for the expectation adjustment, pain coping mechanism, and the rehabilitation plan preoperatively. A team consisting of surgeons, nurses, physical therapists should discuss the treatment strategy with the legal guardians thoroughly. Although preoperative education reduces the level of anxiety in parents, it does not reduce the duration of hospital stay significantly, lower the VAS score and the incidence of complications [26].

Multimodal analgesia is advocated to boost the effect of pain alleviation and reduce the incidence and severity of adverse effects. One of the key aspects of the ERAS program is to reduce the dose of opiates and even eliminate the application of opioids postoperatively [27]. Nonsteroidal anti-inflammatory drugs (NSAIDs) are the cornerstone for postoperative pain alleviation, but the possible sides effects to the digestive and cardiovascular system should not be overlooked. Dexibuprofen, a non-selective NSAID, has been widely used in pediatric orthopaedics to cope with mild to moderate pain, but the long-term effects remain to be investigated. Unlike adults, most children are not able to endure pain. A novel drug such as bupivacaine liposome with longer half-life has been used in developed and used in clinical practice [28]. However, its application in children has not been thoroughly investigated. Dexamethasone is a strong anti-inflammatory drug, high dosage (> 0.1 mg/kg) is able to alleviate all kinds of postoperative pain in adults [29]. De Oliveira GS et al. performed a meta-analysis of randomized controlled trials to evaluate the dose-dependent analgesic effects of perioperative dexamethasone. They reported that an intraoperative infusion of 10 mg mg dexamethasone could reduce the duration of hospital stay significantly, and it is not associated with delayed healing and infection [30]. Dexamethasone has been used as adjuvant to Bupivacaine in the pediatric population [31, 32], but the safety and validity of the intravenous application of glucocorticoids in children for pain management remains to be investigated and rarely used in our institute.

Peripheral nerve block is routinely performed in patients with long bone fracture surgeries; however, it is not commonly performed in DDH patients. Local anaesthetic agents such as lidocaine and ropivacaine have also been reported to use around the incision site for postoperative pain management. As for joint replacement surgeries, surgeons advocate the injection of a cocktail consisting of opiates, NSAIDs, and steroids periarticularly [33]. However, this technique has not been used and validated in our institute for the pediatric population.

American Society of Anesthesiology has recommended that the preoperative fasting for children should be at least 2 h for clear water, 4 h for breast milk, 6 h for non-human or cow milk, 8 h for meat or oily food. It has been validated that 2 h fasting for clear water intake is safe in children, and it significantly reduces the thirst and hunger of sick children while waiting for the surgery [34]. In our institute, we try to calculate the precise timing of surgery and minimize the fasting time for children.

Most of the orthopaedic procedure does not need the drainage tube [35]; however, it is recommended in the DDH patients as the wounds are covered in Spica cast, and there is always the possibility of blood loss from the osteotomy sites causing postoperative hematoma and infection. In routine ERAS program, all kinds of catheters and drainage tubes are encouraged to remove early to promote early rehabilitation.

Early diet is recommended for DDH patients after anaesthesia awareness. However, most young children are not able to eat regular diet immediately after surgery. Noodles or thin soup was recommended for young children on the first meal postoperatively. Food rich in dietary fiber is recommended for the patient to lower the incidence of constipation. Walking is not possible postoperatively as the hips are immobilized in Spica cast. But early ambulation is always encouraged.

Fever was less in the ERAS group,possibly due to better temperature monitoring and management in the operating room. No patient had postoperative urinary tract infection because of early removal.

So far, there are limited reports of the ERAS program in the field of pediatric orthopaedics, most of our core components in the ERAS protocol were derivatives from research in adults. In clinical practice, the application of multimodal analgesia alleviates the postoperative pain response that increases the surgical cost. The predictive increase of medication cost might be compensated by the reduction of hospital stay; however, that requires multicenter study.

Several limitations exist in our study. First and foremost, it has all the biases of a retrospective study, and a prospective randomized control trial might be more convincing. Secondly, the follow-up was not long enough to validate the findings. Thirdly, there might be a surgeon’s bias as different surgeons of the same team performed all the surgeries; the outcome might be varied as per the surgeon’s experience.

## Conclusion

The ERAS protocol is both safe and feasible choice for pediatric DDH patients undergoing osteotomies, and it shortens the duration of hospital stay. Continuous education and training should be given to the ERAS team, including the surgeon, nurses, anesthesiologist, caregiver, pediatrician to implement the protocols more strictly. Long-term follow up is required to validate the benefits of ERAS application in pediatric orthopaedics.

## Data Availability

This is a report of comparative study. To protect privacy and respect confidentiality, no raw data have been made available in any public repository. The operation reports, imaging studies are all retained as per normal procedure within the medical records of our institution.

## Abbreviations

ERAS: Enhanced recovery after surgery
MIS: Minimal invasive surgery
DCT: Damage control theory
POD: Postoperative day
PONV: Postoperative nausea and vomiting
VAS: Visual analog scale
DDH: Developmental dysplasia of the hip
PO: Pelvic osteotomy
PFO: Proximal femoral osteotomy
